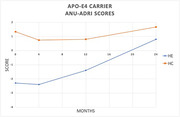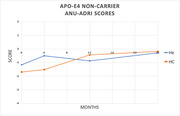# Health Coaching Intervention Retained ANU‐ADRI Scores Over a 2‐year period in APOE e4 Carriers Among Older Adults at Risk for Alzheimer’s Disease

**DOI:** 10.1002/alz.089578

**Published:** 2025-01-09

**Authors:** Michelle Gray, Anthony Campitelli, Ray G Urbina, Megan Jones

**Affiliations:** ^1^ University of Arkansas, Fayetteville, AR USA

## Abstract

**Background:**

The Apolipoprotein epsilon 4 allele (APOE **ε**4) gene is known as the most prevalent genetic risk factor for Alzheimer’s disease (AD). However, many lifestyle risk factors that are modifiable influence the development of AD. Reducing the prevalence of risk factors can prevent or delay up to 33% of AD cases. Health coaching is known to improve health over time in older adults by using a motivational interviewing technique resulting in positive behavior modification. The purpose of this study was to determine if a 2‐year health coaching intervention improves AD risk score among older adults at risk for AD aged 60 and 75 years of age.

**Method:**

Adults between the ages of 60‐75 (*n* = 106) were randomly assigned to a health coaching (HC) or health education (HE) control group. HC met with a health coach once every 4‐6 weeks and HE received biweekly educational emails addressing AD risk factors. Each participant tested at 4 time points: 0 (baseline), 4, 12, and 24 months. At baseline the participants had blood drawn to test for APOE **ε**4 (carrier and non‐carrier). At each time point the participants completed the Australian National University‐Alzheimer’s Disease Risk Index (ANU‐ADRI), a validated tool to predict AD. To determine significant differences between time points for each group and apoe **ε**4 carrier status a mixed factorial ANOVA (α = .05) was used.

**Result:**

Among both carrier groups the HE and HC interventions had no significant mean difference between baseline and 24 months (Non‐carriers HE *p* = .936, HC *p* = .123; Carriers HE *p* = .109, HC *p* = .832). No meaningful difference between non‐carrier HE (‐.881) and HC (‐1.513) or Carrier HC (‐0.333) interventions. Overall, HE risk scores increased over time.

**Conclusion:**

HC intervention retained risk scores among APOE **ε**4 carriers over 2 years among older adults. Within carrier group, HE risk scores had a meaningful change leading to an increased risk of AD. Though HC did not result in an improvement in AD risk, there was not a decline, as we expected. Health coaching has been shown to have a positive effect on AD risk reduction.